# Value of Medical Services

**Published:** 1855-11

**Authors:** 


*Value of Medical Services.*—All things have their price, and some things go very cheap. The greatest sacrifice with which we are familiar, has been lately offered up on the shrine of economy, before that august body, the Board of Supervisors of Erie County. We give the plain figures, confident that they will call forth the earnest sympathies of our readers for the poor sufferer in question.

Dr. J. D. Hill (our amiable friend who is trying to squeeze blood from a turnip by a libel suit against the Buffalo Medical Journal, whereof this is a specimen—how do you like it reader?—if well, dun your neighbor to subscribe!) was, sometime about a year ago, inducted into the Erie County

Penitentiary as Physician thereof. It is understood that he accepted the office principally from motives of patriotism, a fact sufficiently set forth in the figures which follow.

He made (at a distance of about one and a-half miles) 97 visits, and also 1365 prescriptions. So his report informs us. The report of those liberal and high-minded gentlemen, the Commissioners of the Penitentiary, sets forth that they have paid him therefor, the magnanimous sum of $53.25, being at the rate of $00.54 cents and 9 mills per visit! or. $00.03 cents and 9 mills per prescription.

Now we do n't care a straw what price the aforesaid Commissioners may set on this man's services. They probably estimate them as highly as we should. But we do wish to point out to the profession the manner in which these county authorities help and reward a man who took the Penitentiary when not another physician could be induced to accept it at less than the tariff price of our county society. The office went begging for weeks.

Does not this picture of the profits of privateering hold out strong inducements for further violations of the fee bill — *supra sinister?*

To talk seriously: we do fervently exhort our brethren of the Erie County Medical Society to stand by the tariff. Under the present regimé it *does not pay* to violate it, and the signs of the times indicate better things after election. If we possess our souls a little longer, and hold the lash *in terrorem* over any weaker brethren — if such there be — we shall soon have the pleasure of seeing the whole plan of the society fulfilled, and its members recognized as units in an honorable profession, and not as county paupers, set up to be sold to the lowest bidder.

